# Trends in Measures of Childhood Obesity in Korea From 1998 to 2012

**DOI:** 10.2188/jea.JE20140270

**Published:** 2016-04-05

**Authors:** Jinwook Bahk, Young-Ho Khang

**Affiliations:** 1 Institute of Health Policy and Management, Seoul National University Medical Research Center, Seoul, South Korea; 2 Department of Health Policy and Management, Seoul National University College of Medicine, Seoul, South Korea

**Keywords:** body mass index, waist circumference, obesity, child, trends, Korea

## Abstract

**Background:**

During the last several decades, the number of children who are overweight or obese has reached alarming levels worldwide. The purpose of the present study was to examine trends in measures of childhood obesity among Korean children aged 2–19 from 1998 to 2012.

**Methods:**

Height, weight, and waist circumference (WC) were measured, and body mass index (BMI) was calculated. Age-adjusted means of WC and BMI were compared between years. We used three international criteria (International Obesity Task Force [IOTF], World Health Organization [WHO], United States Centers for Disease Control and Prevention [CDC]) and a Korean national reference standard (Korea Centers for Disease Control and Prevention [KCDC]) to calculate age-standardized prevalence of childhood overweight and obesity.

**Results:**

Despite differences in absolute prevalence of childhood overweight and obesity according to the four different criteria, the time trends of prevalence were generally similar across criteria. The prevalence of childhood overweight and obesity generally stabilized from 2001–2012 in both boys and girls. WC decreased from 2001–2012 in both boys and girls aged 2–19.

**Conclusions:**

Further studies exploring the factors causing plateaued trends of childhood obesity measures are needed to implement effective policies for reducing the prevalence of childhood overweight and obesity.

## INTRODUCTION

During the last several decades, the number of children who are overweight or obese has reached alarming levels worldwide.^1^^,^^2^ In 2013, 23.8% of boys and 22.6% of girls in developed countries were overweight or obese.^1^ However, many recent studies from developed countries have reported that the increase in childhood obesity has slowed down; childhood overweight and obesity prevalences have stabilized or sometimes have begun to decline from around the late 1990s and early 2000s,^3^^–^^5^ although these trends vary between genders, age groups, and regions.^6^^–^^8^

Body mass index (BMI) has been commonly used to define childhood overweight and obesity.^9^ However, waist circumference (WC) might better reflect adiposity.^10^ Whether childhood WC is a better predictor of future cardiovascular risks is an unresolved question.^11^^–^^13^ Moreover, compared to studies tracking childhood BMI, relatively few studies have examined the time trends of childhood WC.^14^^–^^18^

Three sets of criteria based on BMI cut-points have been used internationally to assess childhood overweight and obesity: those established by the United States (U.S.) Centers for Disease Control and Prevention (CDC) in 2000, the International Obesity Task Force (IOTF) in 2000, and the World Health Organization (WHO) in 2007.^19^^–^^21^ Several studies from various countries have reported that these three criteria (in addition to their own country’s criteria in some cases) yielded different results for the prevalence of childhood overweight and obesity.^22^^–^^25^ Differences in the prevalence of childhood overweight and obesity according to different criteria are inevitable, considering the differences in reference populations and BMI cut-points (see eFigure 1). However, whether the use of different criteria produces different conclusions about the time trends of childhood overweight and obesity remains unclear. To the best of our knowledge, only one study has examined the time trends of childhood overweight and obesity using different criteria, which showed the prevalence of overweight and obesity stabilized among boys and girls from 2001–2007 in all criteria, while the absolute prevalence of childhood obesity among the criteria were different.^26^

This study is an extension of the above-mentioned study,^26^ which used repeated cross-sectional nationally representative samples of 2- to 19-year-old Korean children and adolescents. In this study, we additionally present changes in WC and BMI Z-score over the years and update the recent prevalence of childhood overweight and obesity from 1998 to 2012, employing four different criteria: those of the IOTF, the U.S. CDC, WHO, and Korea Centers for Disease Control and Prevention (KCDC).

## METHODS

### Study subjects

Data were obtained from five waves of the Korean National Health and Nutrition Examination Survey (K-NHANES) conducted in 1998 (the first wave), 2001 (the second wave), 2005 (the third wave), 2007–09 (the fourth wave), and 2010–12 (the fifth wave). The participants in the K-NHANES were drawn as multi-stage clustered probability samples from Korean households representing the civilian, non-institutionalized population.^27^ The response rates for the K-NHANES were 86.5% in 1998, 77.3% in 2001, 70.2% in 2005, 74.5% in 2007–2009, and 76.5% in 2010–2012. Additional details of the study design and methods are described elsewhere.^27^^,^^28^

A total of 18 174 participants (9493 boys and 8681 girls) aged 2–19 (1808 in 1998, 2940 in 2001, 1986 in 2005, 6022 in 2007–09, and 5418 in 2010–12) were available in the health examination survey, which included anthropometric data for each participant. In the 1998 K-NHANES, we were only able to use data from boys and girls aged 10–19, because the health examination survey including anthropometric measurement had not been conducted among children aged 2–9. Detailed numbers of study subjects by survey year, gender, and age groups are presented in eTable 1. This study was approved by the Seoul National University Hospital Institutional Review Board.

### Anthropometric measurement

Anthropometric measurements (ie, height, weight, and WC) were conducted with the same protocols and anthropometric measurement instruments for all waves of the K-NHANES.^28^ Body weight was measured to the nearest 0.1 kg on a calibrated balance-beam scale while the participants wore a lightweight gown or underwear. Height was measured in the upright position to the nearest 0.1 cm using a stadiometer. WC was measured at the midpoint between the lowest rib and the top of the iliac crest on the mid-axillary line to the nearest 0.1 cm with measurement tape placed horizontal to the floor without indenting the skin. BMI Z-scores were obtained using the method recommended by the U.S. Centers for Disease Control and Prevention.^19^

### Criteria for childhood overweight and obesity

We used four different sets of criteria for childhood overweight and obesity based on BMI measurements: those of the IOTF, U.S. CDC, WHO, and KCDC. The IOTF criteria were adapted based on the extrapolation of adult BMI cut-off points for overweight (25 kg/m^2^) and obesity (30 kg/m^2^) from children living in six countries. We used IOTF cut-points at age 18, but for age 19, we used the same cut-points as for adults (overweight ≥25 kg/m^2^ and obesity ≥30 kg/m^2^), because no specific BMI cut-points have been established for age 19 in the IOTF criteria.^20^ The U.S. CDC growth charts were derived from five national surveys conducted between 1963 and 1994 in the U.S. The U.S. CDC defined overweight as BMI ≥85th percentile and obesity as BMI ≥95th percentile.^19^ The WHO criteria were generated from data collected from the WHO Multicentre Growth Reference Study (MGRS) in six countries.^29^ For ages less than 5, BMI values above +2 standard deviations are considered overweight, and values above +3 SD are considered obese.^30^ For ages 5–19, above +1 SD is equivalent to the overweight cut-points used for adults (≥25.0 kg/m^2^) and above +2 SD corresponds to the cut-points for obesity (≥30.0 kg/m^2^).^21^ The KCDC criteria also used age- and gender-specific BMI values and defined the 85th percentile as the cut-point for “overweight” and the 95th percentile as the cut-point for “obesity”.^31^ The KCDC criteria were based on the National Growth Survey for Korean children in 1997 and 2005, when increase in childhood overweight and obesity might have already begun. eFigure 1 shows the age-specific cut-points for childhood overweight and obesity according to the four sets of criteria.

### Statistical analysis

Analyses were conducted according to sex (boys and girls) and age groups (2–9 and 10–19 years). All statistical analyses were performed using SAS version 9.1 (SAS Institute, Cary, NC, USA). We analyzed changes in the age-adjusted least square mean (standard error) of BMI, BMI Z-score, and WC and presented *P*-values for time trends, using regression analysis (PROC SURVEYREG in SAS) after taking into account the primary sampling units, stratification, and sample weights for K-NHANES. To analyze changes in the age-adjusted prevalence (95% confidence intervals [CI]) of childhood overweight and obesity, we employed the direct standardization method using the 2010 Korean Census population as the standard population. Primary sampling units, stratification, and sample weights were also taken into account in this standardization. In this study, the prevalence of overweight includes the prevalence of obesity. We then used logistic regression analyses (PROC SURVEYLOGISTIC in SAS) to estimate *P* values for trends in the age-adjusted prevalence, after accounting for primary sampling units, stratification, and sample weights of the K-NHANES.

## RESULTS

Table 1 presents trends in the age-adjusted least square mean (standard error) of BMI and BMI Z-score. The increase in BMI among boys aged 2–9 from 2001–2012 was statistically significant (*P* for trend = 0.005). Among boys aged 10–19, the increase in BMI was statistically significant from 1998–2012 (*P* for trend < 0.0001) but not from 2001–2012 (*P* for trend = 0.102). No significant increase in BMI was detected among girls aged 2–9 and 10–19. The increase in BMI Z-score among boys aged 2–9 from 2001–2012 was significant (*P* for trend = 0.011). Among boys aged 10–19, the increase in BMI Z-score from 1998–2012 was statistically significant (*P* for trend = 0.002), while the trend from 2001–2012 was not significant (*P* for trend = 0.090). Meanwhile, a significant decrease in BMI Z-score from 2001–2012 among girls aged 10–19 was observed (*P* for trend = 0.021) (Table 1). eTable 2 provides further detailed information on the time trends in the age-adjusted least square means of body weight and height, showing significant increases in body weight and height among boys and girls aged 10–19 from 1998–2012 and a significant increase in body weight among boys aged 2–9 from 2001–2012.

**Table 1.** Trends in age-adjusted least square mean (standard error) values for BMI and BMI Z-score

	1998	2001	2005	2007–2009	2010–2012	*P* for trend (1998 to 2012)	*P* for trend (2001 to 2012)
**BMI (kg/m^2^)**							
Boys and girls							
Boys and girls, aged 2–19 years		18.52 (0.07)	18.67 (0.09)	18.66 (0.05)	18.64 (0.06)		0.274
Boys and girls, aged 2–9 years		16.61 (0.09)	16.36 (0.09)	16.75 (0.05)	16.74 (0.07)		0.027
Boys and girls, aged 10–19 years	19.88 (0.09)	20.69 (0.11)	20.78 (0.13)	20.49 (0.07)	20.45 (0.08)	0.0004	0.019
Boys							
Boys, aged 2–19 years		18.76 (0.10)	18.85 (0.11)	19.03 (0.07)	18.91 (0.08)		0.117
Boys, aged 2–9 years		16.75 (0.12)	16.46 (0.11)	17.00 (0.07)	17.03 (0.10)		0.005
Boys, aged 10–19 years	19.96 (0.11)	21.09 (0.16)	21.03 (0.18)	20.98 (0.10)	20.76 (0.11)	<0.0001	0.102
Girls							
Girls, aged 2–19 years		18.25 (0.09)	18.48 (0.12)	18.23 (0.07)	18.33 (0.08)		0.950
Girls, aged 2–9 years		16.44 (0.11)	16.25 (0.13)	16.47 (0.07)	16.42 (0.08)		0.702
Girls aged 10–19 years	19.79 (0.12)	20.27 (0.14)	20.50 (0.17)	19.94 (0.10)	20.12 (0.12)	0.398	0.069

**BMI Z score**							
Boys and girls							
Boys and girls, aged 2–19 years		0.17 (0.03)	0.15 (0.04)	0.18 (0.02)	0.15 (0.02)		0.641
Boys and girls, aged 2–9 years		0.23 (0.05)	0.04 (0.05)	0.29 (0.03)	0.26 (0.03)		0.065
Boys and girls, aged 10–19 years	−0.05 (0.03)	0.17 (0.03)	0.21 (0.04)	0.10 (0.02)	0.09 (0.02)	0.012	0.007
Boys							
Boys, aged 2–19 years		0.20 (0.05)	0.20 (0.04)	0.28 (0.02)	0.22 (0.03)		0.463
Boys, aged 2–9 years		0.24 (0.07)	0.09 (0.06)	0.38 (0.03)	0.36 (0.04)		0.011
Boys, aged 10–19 years	−0.05 (0.04)	0.26 (0.05)	0.26 (0.06)	0.23 (0.03)	0.16 (0.04)	0.002	0.090
Girls							
Girls, aged 2–19 years		0.15 (0.04)	0.09 (0.05)	0.06 (0.02)	0.07 (0.03)		0.063
Girls, aged 2–9 years		0.23 (0.05)	−0.02 (0.08)	0.19 (0.04)	0.16 (0.04)		0.958
Girls aged 10–19 years	−0.06 (0.04)	0.07 (0.04)	0.15 (0.05)	−0.04 (0.03)	0.01 (0.03)	0.871	0.021

BMI, body mass index.

Figure shows the distribution of BMI Z-scores among boys and girls aged 2–9 in 2001 and 2000–2012 and those aged 10–19 in 1998, 2001, and 2010–2012. Many of the BMI Z-score distributions were positive among boys and girls aged 2–9. The directionality of the distribution was not as evident among those aged 2–9. Among boys aged 10–19, the distribution in 1998 was in the middle of the BMI Z-scores but shifted toward the right in 2001 and 2010–2012, resulting in a widening of the distribution. However, among girls aged 10–19, no such apparent shifts of the distribution of the BMI Z-score were found across time (Figure).

**Figure.**
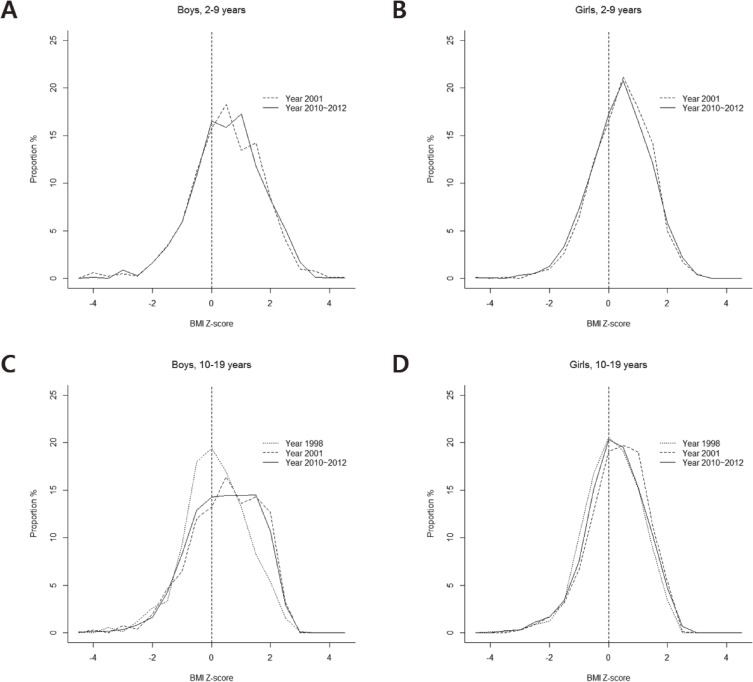
Changes in the distribution of body mass index (BMI) Z-scores among boys and girls, aged 2–9 between 2001 and 2010–2012, and aged 10–19 during 1998, 2001, and 2010–2012.

Table 2 shows trends in the age-adjusted least square means of WC. Significantly decreasing time trends in WC were found from 2001–2012 in both boys (64.54 cm in 2001 and 62.60 cm in 2010–2012) and girls (61.56 cm in 2001 and 59.67 cm in 2010–2012), and in both ages 2–9 and 10–19, while the trends from 1998–2012 among both boys and girls aged 10–19 were not statistically significant. Table 2 indicates that WC at ages 10–19 peaked in 2001 but returned to 1998 levels in 2010–2012. For example, WC among boys was 70.15 cm in 1998, and it increased to 73.48 cm in 2001. Then, the boys’ WC decreased to 72.05 cm in 2005, 71.84 cm in 2007–2009, and 70.74 cm in 2010–2012 (Table 2).

**Table 2.** Trends in age-adjusted least square mean (standard error) values for waist circumference

	1998	2001	2005	2007–2009	2010–2012	*P* for trend (1998 to 2012)	*P* for trend (2001 to 2012)
**Waist circumference (cm)**							
Boys and girls							
Boys and girls, aged 2–19 years		63.13 (0.21)	62.08 (0.23)	61.81 (0.15)	61.21 (0.16)		<0.0001
Boys and girls, aged 2–9 years		55.90 (0.23)	53.94 (0.22)	54.10 (0.16)	53.74 (0.20)		<0.0001
Boys and girls, aged 10–19 years	68.60 (0.27)	70.77 (0.30)	69.82 (0.36)	69.49 (0.21)	68.88 (0.21)	0.350	<0.0001
Boys							
Boys, aged 2–19 years		64.54 (0.28)	63.53 (0.30)	63.45 (0.19)	62.60 (0.22)		<0.0001
Boys, aged 2–9 years		56.36 (0.33)	54.59 (0.32)	54.99 (0.20)	54.72 (0.28)		0.001
Boys, aged 10–19 years	70.15 (0.32)	73.48 (0.45)	72.05 (0.49)	71.84 (0.28)	70.74 (0.31)	0.655	<0.0001
Girls							
Girls, aged 2–19 years		61.56 (0.25)	60.46 (0.30)	59.98 (0.18)	59.67 (0.20)		<0.0001
Girls, aged 2–9 years		55.32 (0.29)	53.22 (0.31)	53.13 (0.19)	52.67 (0.22)		<0.0001
Girls aged 10–19 years	66.99 (0.33)	67.86 (0.34)	67.35 (0.46)	66.84 (0.26)	66.80 (0.30)	0.160	0.009

Table 3 shows trends in the age-standardized prevalence of overweight (including obesity) and obesity using the four different sets of criteria among South Korean boys aged 2–19. Among the four sets of criteria, the WHO MGRS criteria yielded the highest prevalence of overweight and obesity in all age groups across all survey years. The IOTF criteria produced the lowest obesity prevalence in all age categories, while the lowest overweight prevalence was generated by the KCDC criteria. The obesity prevalence according to the WHO MGRS criteria was more than twice the prevalence according to the IOTF criteria in all age groups across all survey years. For example, among boys aged 2–9, obesity prevalence using the IOTF criteria was 5.5% in 2001, 3.1% in 2005, 6.8% in 2007–2009, and 6.4% in 2010–2012, while, using the WHO MGRS criteria, obesity prevalence was 13.1% in 2001, 9.3% in 2005, 14.3% in 2007–2009, and 13.5% in 2010–2012. Although the four sets of criteria resulted in different absolute prevalence values, time trends were similar. No significant trends were found from 2001–2012 in either overweight or obesity prevalence among boys aged 2–9 and 10–19, except for the trend of obesity using the WHO MGRS criteria (*P* for trend = 0.032). Meanwhile, when the 1998 data were included in the time trend analysis, increasing trends of childhood overweight and obesity were statistically significant for all four sets of criteria among boys aged 10–19 (Table 3).

**Table 3.** Trends in age-standardized prevalence (95% confidence intervals) of overweight and obesity among South Korean boys aged 2–19 years

	1998	2001	2005	2007–2009	2010–2012	*P* for trend (1998 to 2012)	*P* for trend (2001 to 2012)
**Boys, aged 2–19 years**							
Obesity							
IOTF		5.6 (4.2–7.1)	4.8 (3.2–6.3)	6.0 (5.1–7.0)	5.4 (4.4–6.4)		0.776
CDC 2000		11.5 (9.6–13.4)	9.5 (7.6–11.4)	11.0 (9.7–12.2)	10.5 (9.1–11.8)		0.783
WHO MGRS		12.7 (10.6–14.8)	10.7 (8.7–12.7)	12.2 (10.9–13.5)	10.9 (9.6–12.2)		0.524
KCDC 2007		8.3 (6.5–10.0)	6.2 (4.4–7.9)	7.7 (6.6–8.8)	7.4 (6.2–8.7)		0.987
Overweight							
IOTF		25.0 (22.1–27.9)	22.7 (19.5–26.0)	25.8 (23.9–27.7)	24.0 (22.0–26.1)		0.981
CDC 2000		28.0 (25.1–30.8)	22.5 (19.6–25.4)	26.1 (24.3–27.9)	25.7 (23.7–27.7)		0.774
WHO MGRS		33.2 (30.3–36.1)	30.1 (26.8–33.4)	33.0 (31.1–35.0)	31.2 (29.1–33.4)		0.786
KCDC 2007		20.4 (17.7–23.1)	17.5 (14.6–20.3)	19.9 (18.2–21.6)	18.4 (16.6–20.3)		0.386

**Boys, aged 2–9 years**							
Obesity							
IOTF		5.5 (3.4–7.7)	3.1 (1.5–4.8)	6.8 (5.4–8.2)	6.4 (4.5–8.2)		0.219
CDC 2000		12.8 (9.5–16.0)	7.8 (5.3–10.3)	12.9 (11.0–14.8)	13.6 (11.2–16.1)		0.341
WHO MGRS		13.1 (9.8–16.4)	9.3 (6.7–11.9)	14.3 (12.3–16.2)	13.5 (11.1–16.0)		0.318
KCDC 2007		7.9 (5.5–10.3)	4.1 (2.3–5.8)	8.7 (7.0–10.3)	8.3 (6.1–10.5)		0.380
Overweight							
IOTF		21.0 (17.1–24.8)	15.2 (12.0–18.5)	21.7 (19.3–24.0)	21.1 (18.2–23.9)		0.450
CDC 2000		28.2 (24.0–32.3)	19.0 (15.6–22.5)	27.4 (24.8–30.0)	27.3 (24.2–30.4)		0.688
WHO MGRS		34.4 (30.0–38.8)	24.3 (20.5–28.1)	34.8 (32.0–37.5)	33.7 (30.5–36.9)		0.478
KCDC 2007		20.2 (16.5–23.8)	12.2 (9.2–15.3)	19.1 (16.7–21.5)	18.1 (15.4–20.8)		0.883

**Boys, aged 10–19 years**							
Obesity							
IOTF	1.9 (1.1–2.7)	5.7 (3.9–7.4)	5.7 (3.5–7.9)	5.6 (4.3–6.9)	4.9 (3.6–6.2)	0.002	0.434
CDC 2000	4.5 (3.2–5.7)	10.8 (8.4–13.1)	10.4 (7.6–13.2)	9.9 (8.2–11.6)	8.7 (7.0–10.3)	0.003	0.115
WHO MGRS	5.0 (3.6–6.4)	12.5 (9.8–15.1)	11.5 (8.6–14.3)	11.0 (9.4–12.7)	9.4 (7.8–11.1)	0.006	0.032
KCDC 2007	3.3 (2.2–4.4)	8.5 (6.2–10.7)	7.3 (4.7–10.0)	7.1 (5.7–8.6)	6.9 (5.4–8.5)	0.011	0.239
Overweight							
IOTF	16.0 (13.4–18.6)	27.2 (23.4–31.1)	27.0 (22.2–31.7)	28.2 (25.6–30.7)	25.7 (23.0–28.3)	<0.0001	0.420
CDC 2000	14.9 (12.6–17.2)	27.8 (24.2–31.5)	24.5 (20.4–28.6)	25.4 (23.1–27.7)	24.8 (22.3–27.3)	<0.0001	0.345
WHO MGRS	21.0 (18.0–24.0)	32.5 (28.9–36.2)	33.4 (28.5–38.3)	32.1 (29.6–34.5)	29.9 (27.2–32.5)	0.0004	0.081
KCDC 2007	11.9 (9.6–14.2)	20.6 (17.0–24.1)	20.4 (16.4–24.4)	20.3 (18.1–22.6)	18.6 (16.2–21.0)	0.001	0.186

CDC 2000, United States Centers for Disease Control and Prevention criteria in 2000; IOTF, International Obesity Taskforce; KCDC 2007, Korea Centers for Disease Control and Prevention criteria in 2007; WHO MGRS, World Health Organization Multicentre Growth Reference Study.

Note: Overweight includes obesity.

Table 4 presents trends in age-standardized prevalence among South Korean girls. The KCDC criteria produced the highest prevalence of childhood obesity, while the IOTF criteria yielded the lowest obesity prevalence among girls in all age groups from 1998–2012. For example, among girls aged 10–19, obesity prevalence using the IOTF criteria was 1.0% in 1998, 1.6% in 2001, 2.6% in 2005, 1.5% in 2007–2009, and 2.7% in 2010–2012, while use of the KCDC criteria resulted in obesity prevalence of 4.7% in 1998, 7.8% in 2001, 7.5% in 2005, 5.8% in 2007–2009, and 7.7% in 2010–2012. The WHO MGRS criteria produced the highest prevalence of childhood overweight among girls in both age groups over the years. The prevalence differences of childhood obesity between the IOTF and the WHO MGRS criteria were greater in girls aged 10–19 (3.7%–6.2%) than in those aged 2–9 (2.3%–3.3%) across the years. The CDC 2000 and the WHO MGRS criteria resulted in similar obesity prevalence for both age groups. For overweight prevalence, the IOTF and the KCDC criteria resulted in similar levels of prevalence among girls aged 2–9, whereas the WHO MGRS and the KCDC criteria resulted in similar overweight prevalence among girls aged 10–19. Trends in overweight and obesity prevalence from 2001–2012 among girls in all age groups were not statistically significant for any of the four sets of criteria. When the 1998 data were included in the time trend analyses, overweight and obesity prevalence from 1998–2012 among girls aged 10–19 showed no significant trends except for obesity prevalence according to the IOTF criteria (*P* for trend = 0.035) (Table 4).

**Table 4.** Trends in age-standardized prevalence (95% confidence intervals) of overweight and obesity among South Korean girls aged 2–19 years

	1998	2001	2005	2007–2009	2010–2012	*P* for trend (1998 to 2012)	*P* for trend (2001 to 2012)
**Girls, aged 2–19 years**							
Obesity							
IOTF		2.4 (1.4–3.4)	3.0 (1.7–4.2)	2.2 (1.6–2.8)	3.1 (2.1–4.0)		0.973
CDC 2000		3.9 (2.6–5.1)	4.5 (3.0–6.0)	4.0 (3.2–4.8)	3.9 (2.9–4.9)		0.785
WHO MGRS		4.3 (3.0–5.6)	4.9 (3.3–6.4)	4.1 (3.2–4.9)	4.6 (3.5–5.7)		0.935
KCDC 2007		7.4 (5.5–9.2)	7.1 (5.1–9.1)	6.1 (5.0–7.2)	7.1 (5.8–8.5)		0.812
Overweight							
IOTF		16.4 (14.0–18.7)	16.7 (14.0–19.4)	15.3 (13.7–16.9)	16.7 (14.8–18.5)		0.915
CDC 2000		17.0 (14.7–19.4)	16.0 (13.4–18.7)	15.4 (13.9–17.0)	16.4 (14.6–18.2)		0.642
WHO MGRS		21.8 (19.3–24.3)	20.7 (17.8–23.7)	20.2 (18.5–22.0)	21.0 (19.0–23.1)		0.448
KCDC 2007		17.4 (15.1–19.7)	18.7 (15.8–21.6)	17.1 (15.4–18.8)	17.5 (15.5–19.4)		0.785

**Girls, aged 2–9 years**							
Obesity							
IOTF		3.7 (1.4–6.0)	3.6 (1.7–5.5)	3.4 (2.2–4.6)	3.7 (2.3–5.1)		0.999
CDC 2000		5.7 (3.0–8.4)	5.8 (3.3–8.3)	6.4 (4.8–8.0)	5.6 (4.1–7.2)		0.763
WHO MGRS		5.3 (2.7–7.9)	6.0 (3.5–8.6)	6.0 (4.5–7.5)	5.9 (4.2–7.6)		0.520
KCDC 2007		6.6 (3.8–9.4)	6.4 (3.6–9.1)	6.7 (5.1–8.3)	6.0 (4.3–7.7)		0.922
Overweight							
IOTF		16.7 (12.8–20.7)	16.9 (12.8–20.9)	17.9 (15.5–20.4)	16.8 (14.2–19.4)		0.830
CDC 2000		20.2 (16.2–24.3)	18.1 (13.8–22.3)	20.9 (18.3–23.4)	19.4 (16.5–22.2)		0.910
WHO MGRS		26.3 (21.8–30.8)	21.4 (17.1–25.7)	26.6 (23.8–29.4)	24.7 (21.6–27.8)		0.903
KCDC 2007		16.9 (12.9–20.9)	16.5 (12.4–20.6)	18.1 (15.7–20.5)	16.4 (13.8–19.0)		0.967

**Girls, aged 10–19 years**							
Obesity							
IOTF	1.0 (0.3–1.7)	1.6 (0.7–2.5)	2.6 (1.0–4.3)	1.5 (0.9–2.1)	2.7 (1.6–3.8)	0.035	0.480
CDC 2000	1.7 (0.8–2.6)	2.8 (1.6–4.1)	3.8 (1.9–5.7)	2.7 (1.8–3.5)	2.9 (1.7–4.1)	0.292	0.457
WHO MGRS	2.1 (1.1–3.1)	3.7 (2.3–5.1)	4.2 (2.2–6.3)	3.0 (2.1–3.9)	3.8 (2.5–5.1)	0.147	0.703
KCDC 2007	4.7 (3.2–6.1)	7.8 (5.4–10.2)	7.5 (4.8–10.2)	5.8 (4.3–7.2)	7.7 (5.9–9.6)	0.068	0.993
Overweight							
IOTF	13.5 (11.3–15.8)	16.2 (13.1–19.3)	16.6 (13.0–20.2)	13.8 (11.8–15.8)	16.6 (14.1–19.0)	0.302	0.965
CDC 2000	11.2 (9.1–13.4)	15.3 (12.2–18.3)	14.9 (11.5–18.3)	12.3 (10.5–14.2)	14.7 (12.4–17.0)	0.200	0.705
WHO MGRS	16.1 (13.6–18.6)	19.2 (15.9–22.5)	20.4 (16.5–24.3)	16.6 (14.5–18.8)	19.0 (16.3–21.6)	0.425	0.617
KCDC 2007	15.6 (13.0–18.2)	17.7 (14.6–20.7)	20.0 (15.9–24.0)	16.5 (14.3–18.7)	18.1 (15.5–20.6)	0.445	0.662

CDC 2000, United States Centers for Disease Control and Prevention criteria in 2000; IOTF, International Obesity Taskforce; KCDC 2007, Korea Centers for Disease Control and Prevention criteria in 2007; WHO MGRS, World Health Organization Multicentre Growth Reference Study.

Note: Overweight includes obesity.

## DISCUSSION

This study examined trends in measures of childhood obesity from 1998–2012 using a representative sample of Korean children aged 2–19. This study is an extension of our prior study^26^ but included more recent data from 2008–2012 and additionally examined trends in WC and BMI Z-score. BMI Z-scores and the changes in the BMI Z-score distribution are useful for assessing a population-wide shift in childhood adiposity over time.^32^

In our calculations of age-standardized prevalences of childhood overweight and obesity, we also took into account primary sampling units and stratification, in addition to sample weights, and used more recent 2010 Korean Census data for standardizing the population, which resulted in slightly different prevalences than were presented in the prior study.^26^

The results of this study showed decreasing trends in WC from 2001–2012 in both boys and girls and for ages 2–9 and 10–19. Time trends in childhood WC have been explored in a few studies, but most of these studies compared only two time points. A study conducted in Switzerland showed a stabilized prevalence trend for the 85th and 95th percentiles of WC among boys and girls aged 6–12 from 1999–2012.^33^ Similarly, decreasing or plateaued trends in WC were observed in Brazilian and Spanish adolescents during the last decade.^18^^,^^34^ In contrast, WC and general obesity prevalence increased among Chinese children and adolescents aged 6–17 from 1993–2009.^17^

Despite the decreasing trends in WC from 2001–2012 in both boys and girls aged 2–9 and 10–19, BMI Z-scores presented different trends by gender and age groups. BMI Z-scores plateaued among boys aged 10–19 and girls aged 2–9, decreased among girls aged 10–19, and increased among boys aged 2–9 from 2001–2012. Considering that body weight and height plateaued from 2001–2012 among boys aged 10–19 and girls aged 2–9 (see eTable 2), these results suggest that lean mass might have increased while central adiposity did not increase among these children from 2001–2012, findings which are consistent with the results for childhood overweight and obesity trends that revealed stabilization from 2001–2012.

In this study, we used four different sets of criteria for the prevalence of childhood overweight (including obesity) and obesity. The IOTF criteria resulted in the lowest prevalence of obesity for both boys and girls at ages 2–9 and 10–19, while the WHO MGRS criteria for boys and the KCDC criteria for girls produced the highest prevalence of obesity among the four sets of criteria. The absolute prevalence differences of childhood obesity were generally the smallest between the WHO MGRS and the CDC criteria compared to the prevalence differences using the other two criteria in both boys and girls and children aged 2–9 and 10–19. In contrast, the IOTF and WHO MGRS criteria produced the biggest differences in obesity prevalence among boys aged 2–9 and 10–19, whereas the IOTF and the KCDC criteria yielded the biggest difference in obesity prevalence among girls aged 2–9 and 10–19. These results from the four sets of criteria were expected, considering their use of different BMI cut-points for childhood obesity and overweight (see eFigure 1).

Despite the inconsistencies in the absolute prevalence of childhood overweight and obesity according to the four sets of criteria, time trends were similar for all four. When different childhood overweight and obesity criteria are used, different prevalences of childhood overweight and obesity will inevitably result. However, it was uncertain how the time trends of childhood overweight and obesity would compare when different cut-points were used. The present findings suggest that analyses using different criteria are not crucial to determine time trends of childhood overweight and obesity. In addition, similar time trends in childhood overweight and obesity would have been produced in other studies^35^^,^^36^ if different criteria were used. When we explored trends of overweight (including obesity) and obesity from 2001–2012, the results showed that the prevalence of overweight and obesity stabilized according to all four sets of criteria among boys and girls, at ages 2–9 and 10–19, except for the trend in obesity prevalence according to WHO MGRS criteria among boys aged 10–19. However, when we examined trends among boys and girls aged 10–19 from 1998–2012, overweight and obesity among boys according to all four sets of criteria and obesity among girls according to the IOTF criteria showed increasing trends. These results suggest that the prevalence of overweight and obesity among boys aged 10–19 might have increased from 1998–2001, and then stabilized from 2001–2012, while the prevalence of overweight and obesity among girls aged 10–19 generally stabilized across all the years surveyed.

A plateaued trend in childhood obesity has been observed in recent years in many developed countries. For example, in England, the prevalence of overweight and obesity among children aged 5–10 increased from 1997–2002 and then stabilized from 2002–2007.^37^ Similarly, stabilized trends have been observed in other countries, such as Australia,^38^ France,^39^ Greece,^40^ and Germany.^41^ Recent plateaued trends of childhood obesity may be explained by public awareness of childhood obesity and interventions at both the individual and policy levels. Stamatakis and colleagues have suggested that massive media attention on obesity and widespread health concerns, as well as policies aimed at reducing obesity, may be related to the stabilization of obesity in England.^37^ Olds and colleagues have indicated that interventions on physical activity and nutrition at the home, school, community, state, and national level resulted in a flattening prevalence of obesity in Australia.^38^ In Korea, Park and colleagues have argued that regular weekly exercise increased from 2001–2005 among Korean adolescents aged 10–19.^42^ Changes in early life nutritional factors in recent decades may also explain plateaued trends in childhood obesity.^43^ Wabitsch et al have noted that childhood obesity rates have stabilized in developed countries and that the possible causes of these trends are the cumulative effects of interventions, such as reducing television viewing duration and consumption of energy-dense foods, as well as increasing daily physical activity.^44^ In addition to the aforementioned explanations, selection bias may play a role in the observed stabilization of obesity rates. As Marild et al have suggested,^45^ if people with a higher prevalence of overweight and obesity have tended to avoid participating in more recent surveys, plateaued or even decreasing prevalences of childhood overweight and obesity may result. Further studies examining these possibilities should be conducted to explain the plateaued or even decreasing prevalence of childhood obesity found in this study.

The present study has several strengths. First, we used five sets of nationally representative data, which contained consistent anthropometric measurements of height, weight, and WC examined by the same protocols and anthropometric measurement instruments from samples of 2- to 19-year-old Korean children. Second, to the best of our knowledge, this is one of the first studies to compare the trends of childhood overweight and obesity using four different sets of criteria. The time trend of childhood obesity according to WHO MGRS criteria showed a significantly decreasing trend among boys aged 10–19, while the other criteria did not present such significant trends. This study also presented dissimilar prevalences of childhood overweight and obesity according to these criteria.

However, our study also has several limitations. First, since K-NHANES began in 1998, we could not examine childhood overweight and obesity trends before 1998. Second, we could not use anthropometric data for children aged 2–9 for the 1998 K-NHANES, because this age group was not included in the first wave of K-NHANES. Third, the possibility of selection bias associated with declining survey participation rates among overweight and obese individuals should be considered in interpretation of the results.

In summary, we examined trends in childhood overweight and obesity in nationally representative samples of South Korean children from 1998–2012 using four different sets of criteria for childhood overweight and obesity. The results indicate that the absolute differences in childhood overweight and obesity prevalence by different criteria were relatively large, although the time trends of these prevalences were generally similar among the criteria. Thus, clarification of the criteria and use of two or more sets of criteria in determining the prevalence of childhood overweight and obesity should be considered when reporting such prevalence measures to the public and monitoring the prevalences and time trends of childhood overweight and obesity to avoid any potential confusion. Moreover, investigations into health outcomes associated with these different cut-off points are of critical importance.^46^ This study showed that the prevalence of overweight and obesity among Korean children stabilized over the last decade and that a decreasing trend in WC was observed. However, there is scarce evidence explaining these recent trends toward stabilization among Korean children. We surmise that increased awareness of childhood obesity, which leads to improved quality of dietary intake and physical activity, could be one possible explanation. Considering the still-high absolute prevalence of childhood overweight, further studies exploring factors causing trends to level off are needed to implement effective policies for lowering rates of childhood overweight and obesity.

## ONLINE ONLY MATERIALS

eTable 1. Number of study subjects by survey year, gender, and age groups.

eTable 2. Trends in least square mean (± standard error) values for weight and height.

eTable 3. Trends in age-standardized prevalence (95% confidence intervals) of overweight and obesity among South Korean boys and girls aged 2–19.

eFigure 1. Comparison of cut-offs for childhood obesity and overweight among criteria proposed by the International Obesity Taskforce (IOTF), United States Centers for Disease Control and Prevention (US CDC), the World Health Organization (WHO), and the Korea Centers for Disease Control and Prevention.
