# Supplementary material for: Trends in Measures of Childhood Obesity in Korea From 1998 to 2012
Source: J Epidemiol. 2016 Apr 5;26(4):199–207. doi: 10.2188/jea.JE20140270 (PMC4808687; doi:10.2188/jea.JE20140270)
Supplement: eTable 1. [file je-26-199-s001.pdf]

eTable 1. Number of study subjects by survey year, gender, and age groups

	1998	2001	2005	2007-2009	2010-2012	Total
**Boys and girls**						
Boys and girls, aged 2-19 years	1,808	2,940	1,986	6,022	5,418	18,174
Boys and girls, aged 2-9 years		1,480	984	2,865	2,524	7,853
Boys and girls, aged 10-19 years	1,808	1,460	1,002	3,157	2,894	10,321
**Boys**						
Boys, aged 2-19 years	917	1,551	1,022	3,149	2,854	9,493
Boys, aged 2-9 years		795	497	1,499	1,338	4,129
Boys, aged 10-19 years	917	756	525	1,650	1,516	5,364
**Girls**						
Girls, aged 2-19 years	891	1,389	964	2,873	2,564	8,681
Girls, aged 2-9 years		685	487	1,366	1,186	3,724
Girls aged 10-19 years	891	704	477	1,507	1,378	4,957
