# Supplementary material for: Trends in Measures of Childhood Obesity in Korea From 1998 to 2012
Source: J Epidemiol. 2016 Apr 5;26(4):199–207. doi: 10.2188/jea.JE20140270 (PMC4808687; doi:10.2188/jea.JE20140270)
Supplement: eTable 2. [file je-26-199-s002.pdf]

eTable 2. Trends in least square mean (standard error) values for weight and height

	1998	2001	2005	2007-2009	2010-2012	P for trend (1998 to 2012)	P for trend (2001 to 2012)
**Body weight (kg)**							
Boys and girls							
Boys and girls, aged 2-19 years		37.92 (0.18)	39.34 (0.27)	38.94 (0.15)	38.70 (0.18)		0.015
Boys and girls, aged 2-9 years		23.68 (0.18)	24.02 (0.17)	24.27 (0.11)	24.11 (0.15)		0.030
Boys and girls, aged 10-19 years	50.77 (0.32)	53.44 (0.35)	53.89 (0.42)	53.31 (0.25)	53.11 (0.28)	<0.0001	0.252
Boys							
Boys, aged 2-19 years		40.00 (0.27)	40.85 (0.38)	41.15 (0.21)	40.73 (0.25)		0.034
Boys, aged 2-9 years		24.09 (0.25)	24.27 (0.22)	24.89 (0.16)	24.87 (0.22)		0.003
Boys, aged 10-19 years	53.08 (0.41)	57.29 (0.55)	56.80 (0.61)	56.92 (0.34)	56.25 (0.39)	<0.0001	0.156
Girls							
Girls, aged 2-19 years		35.64 (0.21)	37.63 (0.33)	36.46 (0.19)	36.45 (0.22)		0.184
Girls, aged 2-9 years		23.18 (0.24)	23.74 (0.25)	23.59 (0.14)	23.29 (0.18)		0.834
Girls aged 10-19 years	48.44 (0.36)	49.38 (0.37)	50.67 (0.51)	49.21 (0.29)	49.62 (0.34)	0.045	0.624
**Height (cm)**							
Boys and girls							
Boys and girls, aged 2-19 years		137.89 (0.21)	139.93 (0.26)	139.08 (0.13)	138.48 (0.17)		0.267
Boys and girls, aged 2-9 years		117.84 (0.18)	119.44 (0.24)	118.62 (0.13)	118.14 (0.15)		0.900
Boys and girls, aged 10-19 years	158.80 (0.25)	159.67 (0.27)	159.96 (0.30)	160.10 (0.17)	160.01 (0.21)	0.0001	0.300

Boys							
Boys, aged 2-19 years		140.11 (0.25)	141.57 (0.33)	141.06 (0.16)	140.65 (0.21)		0.290
Boys, aged 2-9 years		118.27 (0.25)	119.71 (0.29)	119.15 (0.17)	118.81 (0.20)		0.307
Boys, aged 10-19 years	161.80 (0.33)	163.61 (0.36)	163.05 (0.44)	163.40 (0.22)	163.30 (0.27)	0.003	0.668
Girls							
Girls, aged 2-19 years		135.45 (0.31)	138.08 (0.35)	136.84 (0.19)	136.08 (0.22)		0.575
Girls, aged 2-9 years		117.30 (0.24)	119.14 (0.30)	118.03 (0.16)	117.43 (0.21)		0.465
Girls aged 10-19 years	155.77 (0.28)	155.53 (0.29)	156.57 (0.34)	156.35 (0.19)	156.38 (0.21)	0.009	0.043
