# Supplementary material for: Trends in Measures of Childhood Obesity in Korea From 1998 to 2012
Source: J Epidemiol. 2016 Apr 5;26(4):199–207. doi: 10.2188/jea.JE20140270 (PMC4808687; doi:10.2188/jea.JE20140270)
Supplement: eTable 3. [file je-26-199-s003.pdf]

eTable 3. Trends in age-standardized prevalence (95% confidence intervals) of overweight and obesity among South Korean boys and girls aged 2-19

	1998	2001	2005	2007-2009	2010-2012	P for trend (1998 to 2012)	P for trend (2001 to 2012)
**Boys and girls, aged 2-19 years**							
Obesity							
IOTF		4.1 (3.1-5.1)	3.9 (2.8-5.0)	4.2 (3.6-4.8)	4.3 (3.6-5.1)		0.851
CDC 2000		7.9 (6.8-9.0)	7.2 (5.8-8.5)	7.7 (6.9-8.4)	7.4 (6.5-8.2)		0.615
WHO MGRS		8.8 (7.5-10.0)	8.0 (6.6-9.4)	8.3 (7.5-9.1)	7.9 (7.0-8.9)		0.534
KCDC 2007		7.9 (6.6-9.1)	6.7 (5.3-8.0)	6.9 (6.2-7.7)	7.3 (6.3-8.2)		0.846
Overweight							
IOTF		21.0 (18.9-23.0)	19.8 (17.6-22.0)	20.8 (19.5-22.1)	20.5 (19.2-21.9)		0.904
CDC 2000		22.9 (20.9-24.9)	19.4 (17.4-21.4)	21.0 (19.8-22.2)	21.3 (20.0-22.7)		0.564
WHO MGRS		27.9 (25.8-30.0)	25.6 (23.3-27.8)	27.0 (25.6-28.3)	26.4 (24.9-27.9)		0.446
KCDC 2007		19.1 (17.3-20.9)	18.1 (16.0-20.1)	18.5 (17.3-19.8)	17.9 (16.6-19.3)		0.408
**Boys and girls, aged 2-9 years**							
Obesity							
IOTF		4.7 (3.1-6.3)	3.3 (1.9-4.6)	5.2 (4.2-6.1)	5.1 (3.9-6.3)		0.382
CDC 2000		9.6 (7.5-11.7)	6.8 (5.0-8.6)	9.8 (8.5-11.2)	9.8 (8.2-11.4)		0.427
WHO MGRS		9.6 (7.5-11.7)	7.7 (5.9-9.5)	10.3 (9.0-11.7)	9.9 (8.3-11.5)		0.320
KCDC 2007		7.3 (5.5-9.1)	5.0 (3.4-6.7)	7.7 (6.6-8.9)	7.3 (5.8-8.7)		0.563

Overweight							
IOTF		19.1 (16.3-22.0)	16.0 (13.5-18.6)	19.8 (18.0-21.7)	19.1 (17.0-21.3)		0.538
CDC 2000		24.6 (21.7-27.5)	18.5 (15.9-21.2)	24.2 (22.2-26.2)	23.6 (21.2-26.0)		0.912
WHO MGRS		30.7 (27.6-33.9)	22.8 (19.9-25.8)	30.8 (28.7-33.0)	29.4 (26.9-31.9)		0.750
KCDC 2007		18.8 (16.1-21.4)	14.3 (11.7-16.9)	18.6 (16.8-20.4)	17.4 (15.3-19.4)		0.921
**Boys and girls, aged 10-19 years**							
Obesity							
IOTF	1.5 (1.0-2.0)	3.7 (2.6-4.8)	4.3 (2.8-5.8)	3.6 (2.9-4.4)	3.9 (3.0-4.8)	0.0001	0.864
CDC 2000	3.2 (2.4-3.9)	7.0 (5.6-8.3)	7.4 (5.5-9.2)	6.5 (5.5-7.4)	6.0 (5.0-7.0)	0.001	0.088
WHO MGRS	3.6 (2.7-4.5)	8.3 (6.8-9.8)	8.2 (6.2-10.1)	7.2 (6.2-8.2)	6.8 (5.7-7.9)	0.002	0.048
KCDC 2007	3.9 (3.1-4.8)	8.2 (6.6-9.8)	7.6 (5.6-9.5)	6.5 (5.4-7.5)	7.3 (6.1-8.5)	0.002	0.406
Overweight							
IOTF	14.7 (12.9-16.6)	22.0 (19.3-24.8)	21.9 (18.8-25.0)	21.3 (19.7-23.0)	21.4 (19.6-23.1)	<0.0001	0.574
CDC 2000	13.0 (11.3-14.7)	21.9 (19.3-24.4)	19.9 (17.1-22.6)	19.2 (17.7-20.6)	20.1 (18.4-21.7)	<0.0001	0.347
WHO MGRS	18.6 (16.5-20.7)	26.2 (23.5-28.9)	27.2 (24.0-30.3)	24.8 (23.0-26.5)	24.7 (22.9-26.4)	0.001	0.106
KCDC 2007	13.6 (11.8-15.5)	19.3 (16.9-21.6)	20.2 (17.3-23.1)	18.5 (16.9-20.1)	18.3 (16.6-19.9)	0.005	0.191

CDC 2000, United States Centers for Disease Control and Prevention criteria in 2000; IOTF, International Obesity Taskforce; KCDC 2007, Korea Centers for Disease Control and Prevention criteria in 2007; WHO MGRS, World Health Organization Multicentre Growth Reference Study.

Note: Overweight includes obesity.
