# Supplementary material for: Trends in Measures of Childhood Obesity in Korea From 1998 to 2012
Source: J Epidemiol. 2016 Apr 5;26(4):199–207. doi: 10.2188/jea.JE20140270 (PMC4808687; doi:10.2188/jea.JE20140270)
Supplement: eFigure 1. [file je-26-199-s004.pdf]

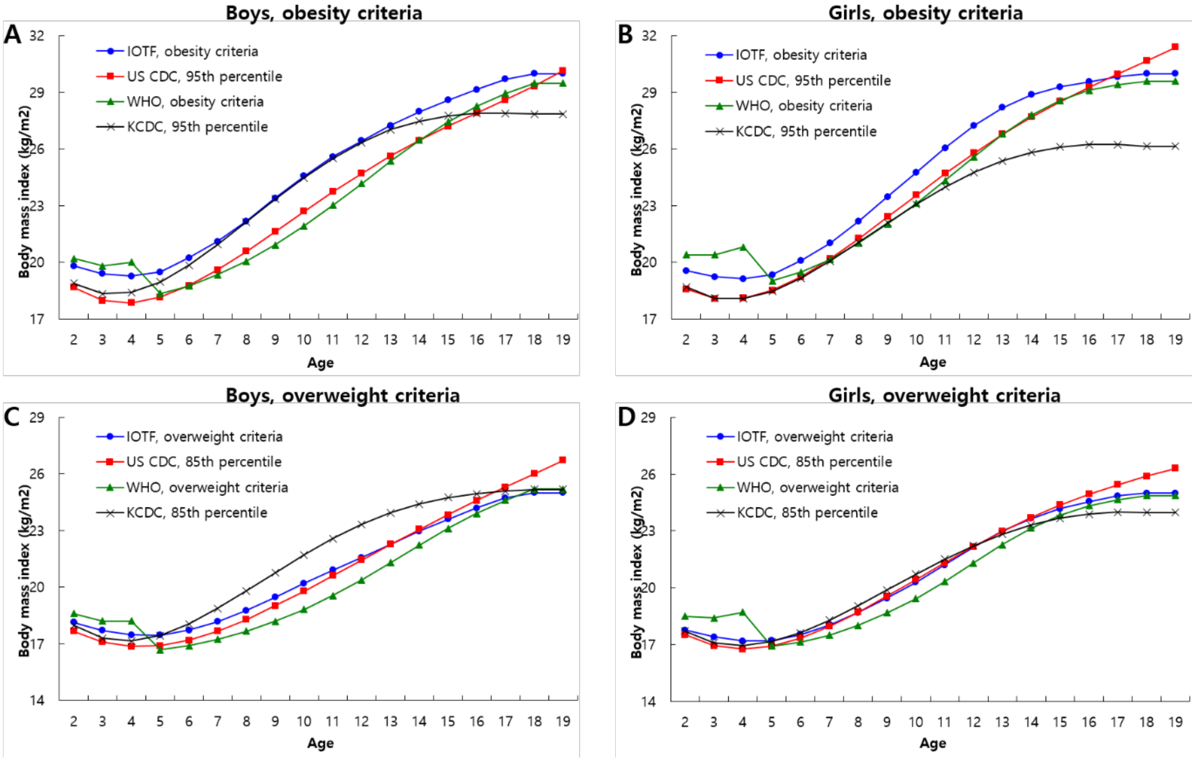

eFigure 1. Comparison of cut-offs for childhood obesity and overweight among criteria proposed by the International Obesity Taskforce (IOTF), United States Centers for Disease Control and Prevention (US CDC), the World Health Organization (WHO), and the Korea Centers for Disease Control and Prevention.

Note: The US CDC criteria for the “overweight” and the “at risk of overweight” categories were considered to be obesity and overweight criteria, respectively, for the comparison with other criteria. Different cut-offs (based on standard deviation [SD] in body mass index) were used for ages under 5 (+2 SD for overweight and +3 SD for obesity) and for the ages of 5-19 (+1 SD for overweight and +2 SD for obesity) in the WHO criteria. The figures were also presented in our prior paper (Khang & Park, Int J Pediatr Obes 2011).

Reference

Khang YH, Park MJ. Trends in obesity among Korean children using four different criteria. Int J Pediatr Obes. 2011;6:206-14.
